# A cyclic vertical deviation with dysthyroid ophthalmopathy: a case report

**DOI:** 10.1186/s12886-016-0300-7

**Published:** 2016-07-22

**Authors:** Ji-Sun Paik, Suk-Woo Yang, Shin Hae Park

**Affiliations:** Department of Ophthalmology and Visual Science, Seoul St. Mary’s Hospital, College of Medicine, The Catholic University of Korea, 222 Banpo-daero, Seocho-Gu, Seoul, 137-701 South Korea

**Keywords:** Cyclic vertical deviation, Dysthyroid ophthalmopathy, Alternating diplopia, Alternating orthotropia

## Abstract

**Background:**

Cyclic strabismus is a very rare condition of ocular motility characterized by alternating strabismus and orthotropia. We report a patient with a 48-h cycle of vertical deviation associated with dysthyroid ophthalmopathy that spontaneously resolved.

**Case presentation:**

A 40-year-old woman experienced sudden onset vertical double vision. She had already been diagnosed with hyperthyroidism. Asymmetric enlargement of the inferior and medial rectus muscles was demonstrated in her left eye on computed tomography, and was compatible with dysthyroid ophthalmopathy. She clearly described the “every-other-day” pattern of diplopia that spontaneously switched around 8 o’clock every night. She exhibited left hypotropia of 35 prism diopters on a bad day, and 7 prism diopters on a good day.

**Conclusions:**

This case illustrates that cyclic vertical deviation may spontaneously resolve along with the course of the underlying diseases that initiated the cyclic deviation, especially in patients with conditions involving motor component changes such as dysthyroid ophthalmopathy.

## Background

Cyclic strabismus is a rare disorder of ocular motility characterized by alternating strabismus and orthotropia. It may occur spontaneously in childhood [1], in adults, however, it is mostly associated with acquired pathological conditions such as central nervous system lesions, underlying visual deficits, and ocular motility disorders [2]. Almost all of these types of strabismus involve cyclic esotropia, but a few cases of isolated vertical deviation have been reported [3–5]. Many studies have attempted to clarify the pathophysiology of this condition, but the precise mechanisms remain unknown. Disruption of the innate biological clock and involvement of the suprachiasmatic nucleus of the hypothalamus are two proposed mechanisms [2]. A relationship between cyclic vertical deviation and ocular neuromyotonia was recently suggested with similar neurophysiological mechanisms [4]. We herein present a patient with a 48-h cycle of vertical deviation associated with dysthyroid ophthalmopathy that spontaneously resolved.

## Case presentation

**Fig. 1:**
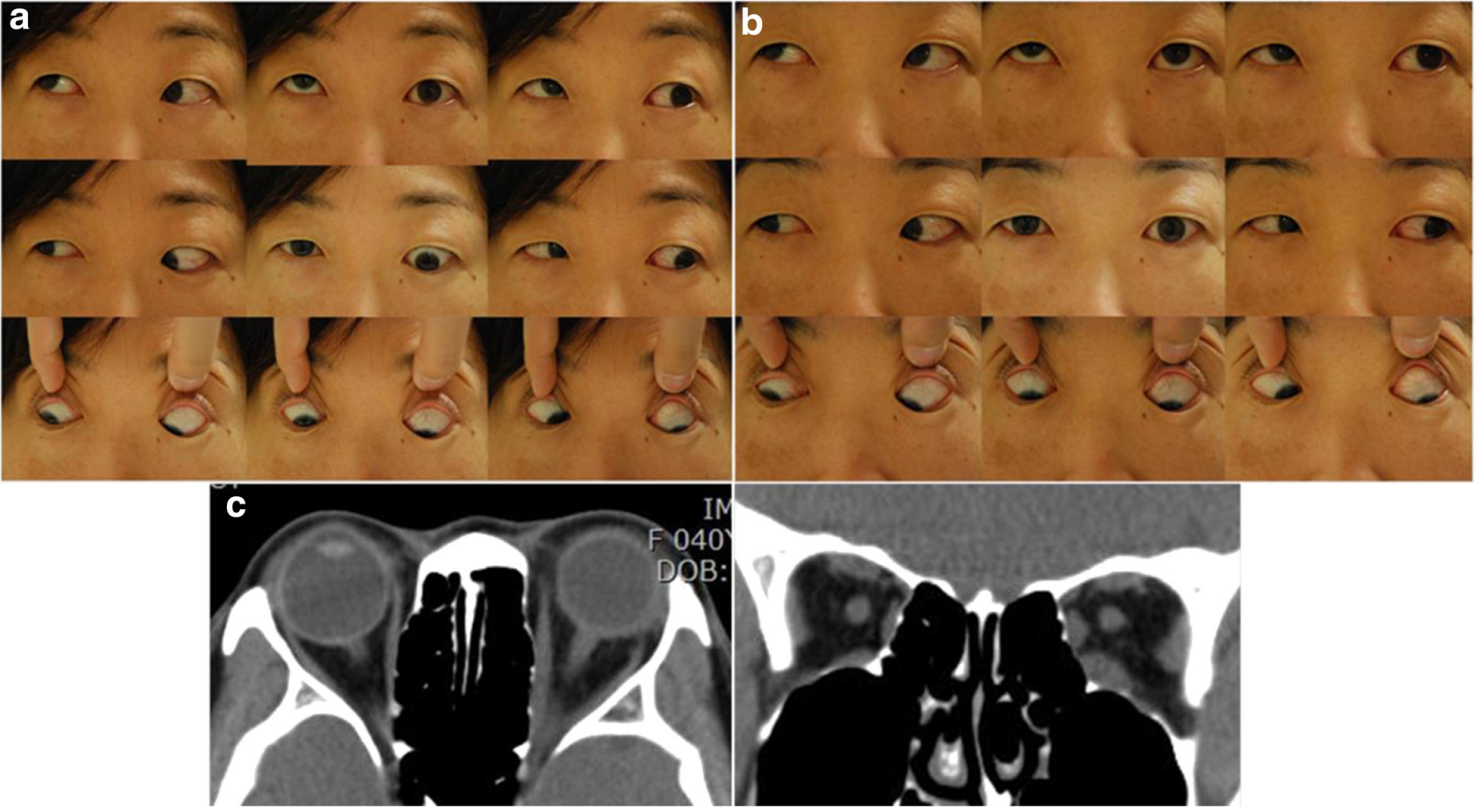
Patient’s photographs on a “bad” day and a “good” day, plus computed tomography (CT) scans. **a** “Bad” day. The patient manifested a 35 prism diopters (PD) left hypotropia in the primary position with marked limitation of elevation in her left eye. **b** “Good” day. The patient had 7 PD left hypotropia in the primary position with mild limitation of elevation in her left eye. **c** A 2-mm sized left exophthalmos and asymmetric enlargement of the inferior and medial rectus muscles were present in her left eye on a CT scan

A 40-year-old woman was referred to our strabismus clinic with a complaint of double vision. She had already been diagnosed with hyperthyroidism, and her thyroid function had normalized after 2 months of treatment with antithyroid medication; subsequently, 2.5 mg carbimazole was used as a maintaining dose. She did not take systemic steroid medication, because her dysthyroid ophthalmopathy was not severe (Clinical Activity Score, CAS 2) and there was no aggravated symptomatic sign. She subsequently developed worsening diplopia and proptosis in her left eye 1 month prior to presentation. At the initial examination, her visual acuity was normal in both eyes (20/20), and 2 mm exophthalmos was noted in her left eye. She exhibited left hypotropia of 35 prism diopters (PD) in the primary position with marked limitation of left eye elevation (Fig. 1a). Asymmetric enlargement of the inferior and medial rectus muscles was demonstrated in her left eye on computed tomography (CT), and was compatible with dysthyroid ophthalmopathy (Fig. 1c). The forced duction test (FDT) revealed restriction in the upward direction in her left eye. She described a highly predictable fluctuating pattern of diplopia, that spontaneously switched at approximately 8 o’clock every night. To differentiate the causes of the fluctuating diplopia, such as myasthenia gravis, we performed laboratory tests, including a determination of acetylcholine receptor antibody titer; all results were normal. Based on her description, we recommended that she keep a diary to record her diplopia episodes and have her picture taken during episodes of both mild and severe diplopia. At the next visit, during which she exhibited relatively mild diplopia, cover testing revealed left hypotropia of 7 PD in the primary position with mild limitation of left eye elevation (Fig. 1b). Her diary clearly revealed the “every-other-day” pattern of diplopia. During the 6 months since the first visit, her thyroid function remained normal and the proptosis of her left eye decreased. A cycle involving diplopia-free days was gradually predominant. Finally, the left hypotropia of 7 PD remained, but without a cyclic pattern. Her diplopia was well compensated with mild chin elevation.

## Conclusions

No mechanism for cyclic strabismus has been determined, although diurnal and circadian rhythms or biological clock mechanisms are assumed to play a role [1, 2, 6]. Diurnal variation and circadian rhythms occur normally. Cyclic deviations associated with acquired pathophysiological conditions can be subdivided into those involving central nervous system lesions (head trauma, high fever, cerebrovascular accidents, severe infection, encephalitis, tumors, or seizures) and those involving peripheral lesions. Peripheral lesions can be differentiated into those involving primarily motoric changes in peripheral structures (e.g., squint surgery or ocular myositis), and those involving sensory disturbances (e.g., visual loss or lens loss) [3].

In the present case, enlargement of the extraocular muscles associated with dysthyroid ophthalmopathy resulted in disruption of binocular fusion, which may have acted as a trigger event for the onset of the cyclic vertical deviation. Interestingly, the patient’s deviation was initiated and became aggravated with the onset of active dysthyroid ophthalmopathy. During the follow-up period, it decreased and subsided along with spontaneous remission of her thyroid eye disease and normalization of her thyroid hormonal level without any medical or surgical management. Roper-Hall et al. reported that cyclic vertical deviation could be a possible forme fruste of ocular neuromyotonia with similar neurophysiological mechanisms [4]. Ocular neuromyotonia is characterized by brief involuntary episodic contractions of ocular muscles, with a sustained tonic contraction, with episodes lasting from seconds to 3–4 min [7]. Cyclic vertical deviation has similar clinical characteristics to ocular neuromyotonia except that the sustained strabismic phase lasts for 24 h on an alternate day cycle [4]. The patient had a spontaneously switching “every-other-day” pattern of diplopia, compatible with cyclic strabismus. Further studies with more cases are needed to confirm cyclic vertical deviations as a variant form of ocular neuromyotonia and to investigate treatment efficacy with membrane stabilizing agents to control abnormal muscle spasms.

The occurrence of cyclic vertical deviation associated with dysthyroid ophthalmopathy has been reported in four adults since the first description by Knapp in 1979 [8]. All patients were treated by recession of the inferior rectus muscle. Based on the hypothesis that cyclic deviation is a strabismic condition with a “burst of orthophoria”, surgical treatment of the full amount of heterotropia on “strabismic” days has been tried. This treatment successfully terminates the cycle without overcorrection on “non-strabismic” days in most patients [3–5]. However, surgical treatment for cyclic strabismus should be considered carefully after a long follow-up [4, 9]. Our findings provide additional evidence that cyclic vertical deviation may spontaneously resolve, along with the course of the underlying diseases that initiated the cyclic deviation, especially in patients with conditions involving motor component changes such as dysthyroid ophthalmopathy.

## Abbreviations

CAS, clinical activity score; CT, computed tomography; FDT, forced duction test; PD, prism diopters.
